# Effectiveness and safety of systemic therapy for moderate-to-severe atopic dermatitis in children and adolescent patients: a systematic review

**DOI:** 10.3389/fimmu.2024.1367099

**Published:** 2024-05-15

**Authors:** Yu Zheng, Rui-Lian Ding, Jin Bu

**Affiliations:** Hospital for Skin Disease (Institute of Dermatology), Chinese Academy of Medical Sciences and Peking Union Medical College, Nanjing, Jiangsu, China

**Keywords:** effectiveness & efficiency (E&E), safety, children, adolescent, systemic therapy, atopic dermatitis (AD)

## Abstract

**Importance:**

Due to comorbidities and associated safety risks, the management of severe atopic dermatitis (AD) in pediatric and adolescent patients poses significant challenges.

**Objective:**

To examine the efficacy and safety of systemic therapies for the treatment of moderate-to-severe atopic dermatitis in children and adolescents.

**Evidence review:**

On Feb 29, 2024, a systematic literature search was conducted in Embase, PubMed, and the Cochrane Central Register of Controlled Trials (Central). No date restrictions were applied. Randomized clinical trials, cohort studies, large case series, and meta-analyses were assessed to evaluate the efficacy (or effectiveness) and/or safety of systemic treatments for moderate-to-severe atopic dermatitis in children and adolescents.

**Findings:**

A preliminary search yielded 1457 results, from which 19 unique articles with a total of 3741 patients were included in the analysis. Overall, the available data for each systemic medication are limited, and the overall quality of the included studies on conventional systemic treatments is relatively low. When Dupilumab was used as a standalone treatment, 30%-40% of infants and toddlers aged 6 months to 2 years achieved EASI-75, while 50% of patients aged 2 to 6 years achieved EASI-75. In children aged 6 to 12 years, 33.0%-59.0% of atopic dermatitis patients achieved EASI-75, and when combined with topical corticosteroids (TCS), 69.7%-74.6% achieved EASI-75. Long-term data showed EASI-75 rates ranging from 75.0% to 94.0% for this age group. For adolescents aged 12 to 18 years, 40%-71% of patients achieved EASI-75 within 12 to 16 weeks, and by week 52, 80.8% of patients achieved EASI-75.Abrocitinib treatment resulted in 68.5%-72.0% of patients achieving EASI-75. Omalizumab treatment at week 24 showed a percentage change in SCORAD scores of -12.4%. In the Methotrexate treatment group, there was a SCORAD change of -26.25% at week 12, while the Cyclosporine A group had a SCORAD change of -25.01%. Patients treated with IVIG (Intravenous Immunoglobulin) showed a -34.4% change in SCORAD percentage scores at week 4, which further decreased by 47.12% at week 24. Patients receiving 4mg of Baricitinib and TCS had a 52.5% rate of EASI-75 at 16 weeks, and patients receiving different doses of upadacitinib had a 63-75% rate of EASI-75 at 16 weeks. The rate of EASI-75 at 16 weeks was around 28% in patients who received various doses of Tralokinumab.The most common adverse events observed were nasopharyngitis, respiratory events and dermatitis atopic.

**Conclusions and relevance:**

Awareness of adverse events and concomitant medications is crucial, and appropriate dosing and frequent laboratory and clinical monitoring are also essential. More real-world evidence and prospective cohort studies analyzing the effectiveness and safety of systemic therapies in children and adolescents are of paramount importance for optimizing personalized, effective, and safe management of the growing population of patients with atopic dermatitis in this age group.

## Introduction

Atopic dermatitis is a chronic disorder that usually starts in childhood but often persists in adulthood (1). If atopic dermatitis is inadequately controlled with emollient use and topical anti-inflammatory therapies, systemic medications including ciclosporin, azathioprine, and methotrexate can be used to treat children with moderate-to-severe disease (2). However, systemic therapies are rarely used in children because the natural disease course often leads to spontaneous improvement, and because their use requires continuous blood monitoring and is associated with tolerance issues and modest efficacy (3). Moreover, oral medication can be challenging to use in children. Accordingly, there is a need for systemic therapies that are efficacious and have a favorable safety profile. As a result, many dermatologists seem to take a cautious approach when treating this population, which can lead to undertreatment. The objective of this systematic review was to systematically evaluate the available evidence on the efficacy or efficacy and safety of systemic anti-atopic dermatitis treatment in children and adolescents.

## Methods

### Search strategy

This Systematic reviews were conducted and reported in accordance with the Cochrane Handbook of Systematic Reviews and the Preferred Reporting Project for Systematic Reviews and Meta-Analyses (PRISMA) reporting guidelines (4, 5). A systematic literature search was conducted on 29 February 2024 in Embase, PubMed and the Cochrane Central Register of Controlled Trials (Central). With the support of the Medical Librarian, we combined all relevant synonyms for atopic dermatitis and adolescents and children with all currently available conventional and modern systemic anti-atopic dermatitis treatments (**Supplementary eTable 1**). There is no use date limit. The list of references included in the article was screened for other relevant research.

### Study selection

Qualification assessment, data extraction, quality assessment and bias risk assessment were carried out independently by 2 reviewers (Yu Zheng and Rui-Lian Ding). In case of discrepancies, the third examiner (Jin Bu). These include randomized clinical trials (RCTs), cohort studies, large case series (total ≥ 8 patients), and meta-analyses evaluating efficacy, efficacy, and/or safety in patients 18 years of age and younger with atopic dermatitis. To provide a complete overview, additional studies may be included in cases where two reviewers agree on the relevance of the article, for example, in cases where different cutoff values are used, or where a relatively older population is included in the study. Studies in languages other than English were excluded, as were case reports, small case series (total <8 patients), conference abstracts, oral exchanges, and expert opinions. If the full text of the original text was not available or more information was requested, at least one attempt was made to contact the author of the original text.

### Outcome measures

The primary outcome was efficacy or effectiveness (for ease of reading, both of the following are expressed as effectiveness), assessed by the percentage change of 75% (EASI75) in the eczema Area and Severity Index in adolescents and children at 12、16、24、36 weeks and the percentage decrease in AD score (SCORAD). Secondary outcome measures were EASI50, EASI90 at weeks 12 to 16 and EASI75 at week 4、24、36, as well as long-term efficacy, and treatment-related safety and tolerability.

### Data extraction and quality assessment

Data were extracted using a predesigned form. Percentages were calculated by the reviewers wherever possible, if not stated in the articles. Study quality was graded according to the Strengthening the Reporting of Observational Studies in Epidemiology (STROBE) reporting guideline for observational studies (6) and the Consolidated Standards of Reporting Trials (CONSORT) reporting guideline for RCTs (7). Risk of bias was assessed using the Newcastle-Ottawa Scale for cohort and case-control studies (8) and the Cochrane Risk of Bias Tool for RCTs (5). P <.05 indicated significance.

## Results

### Study characteristics

**Figure 1:**
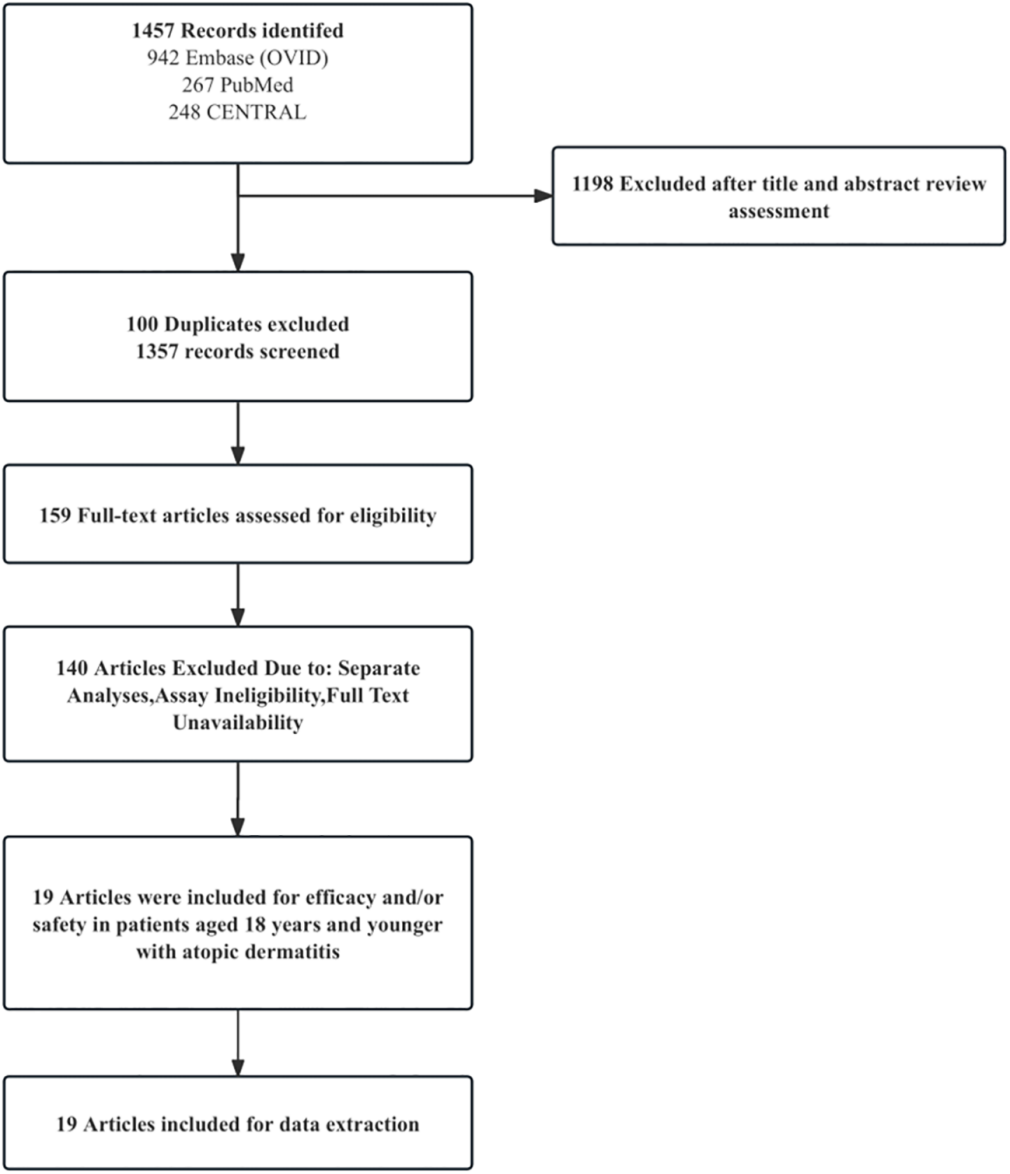
Flow Diagram of the literature Search.

**Figure 2:**
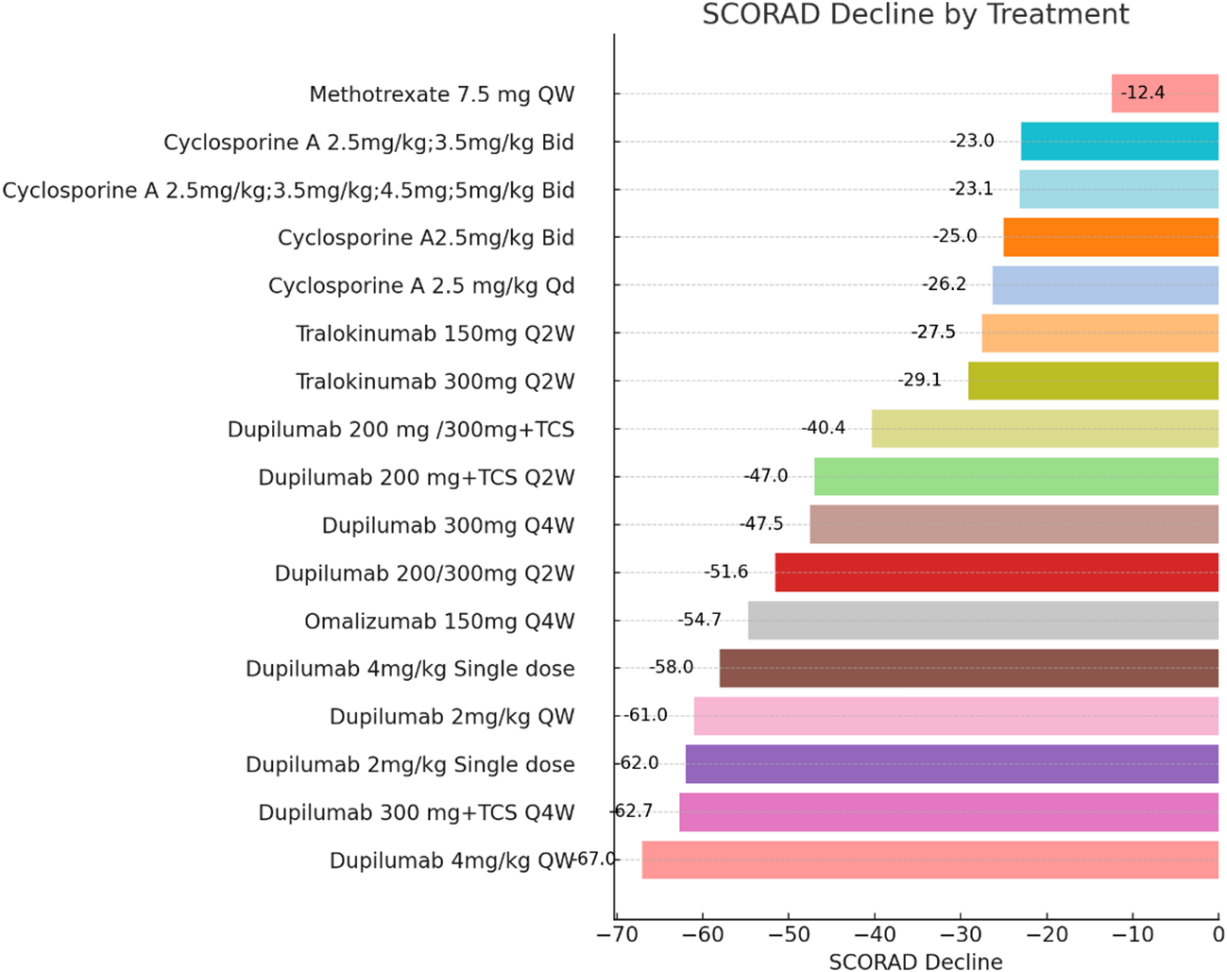
Efficacy or Effectiveness in Children and Adolescent Patients at Induction Phase (Weeks 12-16). Each bar chart represents the change in SCORAD (Scoring Atopic Dermatitis) scores in pediatric and adolescent patients following the administration of systemic medications for atopic dermatitis. Patients with different treatment durations were excluded from this figure. The dataset exhibited significant heterogeneity, precluding a proper meta-analysis. The drugs IVIG and Abrocitinib were not included in the analysis presented in this figure. QW, quaque week; Q2W, quaque two weeks; Q4W, quaque quattuor weeks; Bid, bis in die; Qd, quaque die; TCS, Topical Corticosteroid.

**Figure 3:**
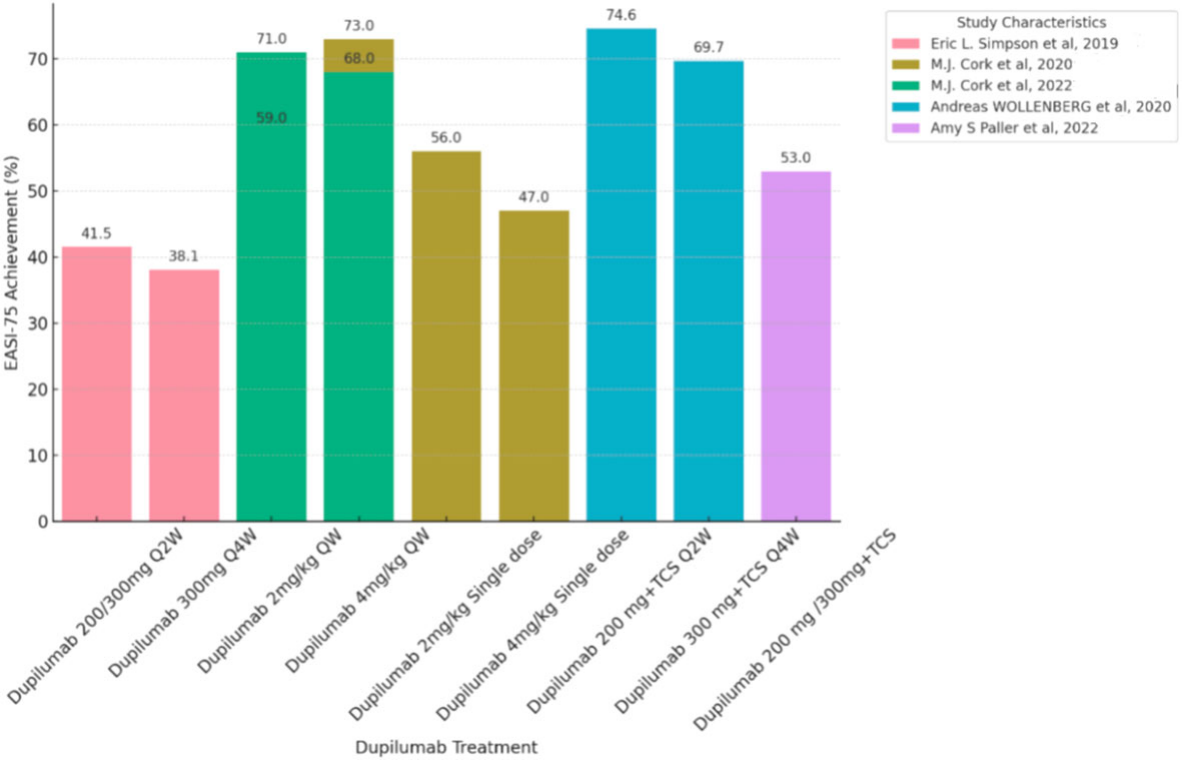
Efficacy or Effectiveness of Dupilumab in children and adolescents during induction period (12-16 weeks). Each bar chart represents a percentage change of 75% in the eczema area and Severity Index (EASI75) in pediatric and adolescent patients treated with Dupilumab. Patients with different treatment duration and who did not use the EASI-75 score were excluded from this chart. The dataset showed significant heterogeneity, so a proper meta-analysis was not possible. QW, quaque week; Q2W, quaque two weeks; Q4W, quaque quattuor weeks; TCS, Topical Corticosteroid.

A literature search yielded 1457 unique articles, including 19 reporting the efficacy and safety of systemic anti-atopic dermatitis treatment in children and adolescents (**Figure 1**). Six studies (31.5%) showed a higher risk of selection bias, and the overall quality of modern systemic therapy studies was higher than that of conventional therapy studies (**Supplementary eTables 3**-**4**). No studies have evaluated the effectiveness and/or safety of other drugs such as Rituximab, Lebrikizumab, Nemolizumab and Spesolimab in children and adolescent patients. **Tables 1**, **2** summarize the efficacy of systemic anti-atopic dermatitis treatments in children and adolescents, alongside study outcomes, quality, and risk of bias. **Figures 2**, **3** give a comparison of the efficacy of treatment modalities in children and adolescents.

**Table 1:** The efficacy or effectiveness of systemic anti-atopic dermatitis treatment in children and adolescents was included in the data extraction studies^a^.

	Source and Study design	No.of entry/included/ finished OLE	Duration of OLE	Treatment	Time	Dose	Frequency	Age,y/m		Baseline EASI	Objective-SCORAD	No. of patients	
								Cutoff,y/m	Mean (SD) [range],y/m	mean (SD) [range]	mean (SD) [range]	Aged ≥12 y	Aged <12 y
1	Eric L. Simpson et al, 2019, (9), RCT-Phase3			Dupilumab	16wk	200mg^b^	Q2W	12,y	13.8 (1.63)[12-18]	36.1(14.62)[NR]	57.4(12.21)[NR]	43	NA
						300mg^b^	Q2W	12,y	15.2 (1.61)[12-18]	34.4(13.05)[NR]	57.3(10.92)[NR]	39	NA
						300mg^b^	Q4W	12,y	14.4 (1.6)[12-18]	35.8(14.8)[NR]	69.8(14.1)[NR]	84	NA
				Placebo				12,y	14.4 (1.8)[12-18]	35.5 (14.0)[NR]	70.4 (13.3)[NR]	85	NA
2	Andrew Blauvelt et al (10), 2022, RCT-Phase3, OLE	294/102/102	52wk	Dupilumab	52wk	300mg^b^	Q4W	12,y	13.6 (1.75) [12-18]	11.5 (13.0)[NR]	28.0 (15.17)[NR]	294	NA
3	M.J. Cork et al, 2022 (11),RCT-Phase2			Dupilumab	12wk	2mg/kg	QW	12,y	15(2)[12-18]	35(17)[NR]	68(13)[NR]	20	NA
				Dupilumab	12wk	4mg/kg	QW	12,y	14(2)[12-18]	29(15)[NR]	63(14)[NR]	20	NA
4	M.J. Cork et al, 2022 (11),RCT-Phase3, OLE	40/36/34	52wk	Dupilumab	52wk	2mg/kg	QW	12,y	15(2)[12-18]	26(17)[NR]	56(17)[NR]	17	NA
				Dupilumab	52wk	4mg/kg	QW	12,y	14(2)[12-18]	21(18)[NR]	54(24)[NR]	19	NA
5	A.S. Paller et al, 2020 (12),RCT-Phase2, OLE	40/NR/NR	52wk	Dupilumab	16wk	3mg/kg	Single Injection	6,m	15.4 (6.96)[6-24],m	35.2(9.21)[NR]	69.8 (13.10)[NR]	NA	10
						6mg/kg	Single Injection	6,m	15.9 (5.51)[6-24],m	40.2(11.81)[NR]	75.9 (11.74)[NR]	NA	10
						3mg/kg	Single Injection	24,m	45.8 (16.13)[24-76],m	34.4(14.25)[NR]	73.5 (10.20)[NR]	NA	10
						6mg/kg	Single Injection	24,m	53.2 (11.23)[24-76],m	35.1(12.94)[NR]	75.1 (8.08)[NR]	NA	10
6	M.J. Cork et al, 2020 (13),RCT-Phase2			Dupilumab	12wk	2mg/kg	Single dose for 8 wk followed by once weekly for 4 wk	10,y	8(2)[6–11]	33(16)[NR]	66(13)[NR]	NA	18
						4mg/kg	Single dose for 8 wk followed by once weekly for 4 wk	10,y	8(2)[6–11]	39(19)[NR]	73(13)[NR]	NA	19
7	M.J. Cork et al, 2020 (13),RCT-Phase3, OLE	38/33/33	52wk	Dupilumab	52wk	2mg/kg	QW	6,y	9(2)[6–11]	21(18)[NR]	52(17)[NR]	NA	17
				Dupilumab		4mg/kg	QW	6,y	8(2)[6–11]	32(20)[NR]	67(18)[NR]	NA	16
8	Andreas WOLLENBERG et al, 2020 (10), RCT-Phase3			Dupilumab 200 mg+TCS	16wk	200 mg^b^	Q2W	6,y	9.5 (1.36)[6-12]	37.1 (11.77)[NR]	58.5 (9.00)[NR]	NA	59
				Dupilumab 300 mg+TCS	16wk	300 mg^b^	Q4W	6,y	8.5 (1.74)[6-12]	37.4 (12.45)[NR]	61.0 (9.63)[NR]	NA	122
				Placebo+TCS	16wk		Q2W/Q4W	6,y	8.3 (1.76)[6-12]	39.0 (12.01)[NR]	59.5 (10.12)[NR]	NA	123
9	Andreas WOLLENBERG et al, 2020 (10), RCT-Phase3, OLE		52wk	Dupilumab 300 mg(+TCS)	52wk	300 mg^b^	Q4W	6,y	8.6 (1.75)[6-12]	12.3 (15.13)[NR]	29.3 (17.81)[NR]	NA	146
10	Andrew Blauvelt et al, 2020 (14), OLE	299/106/104	52wk	Dupilumab	16wk	2mg/kg/wk;4mg/kg/wk	QW	12,y	NR(NR)[12-18]	NR	NR	104	NA
11	L. Stingeni et al, 2020 (15), Prospective			Dupilumab	16wk	200mg/300mg^b^	Q2W/Q4W	12,y	15.1(1.5)[12-18],y	26.2(7.7)[NR]	NA	139	NA
12	L. Stingeni et al, 2022 (16), Prospective			Dupilumab	52wk	200mg/300mg^b^	Q2W/Q4W	12,y	NR(NR)[12-18]	NR	NA	121	NA
13	Angel D. Paganet al, 2022 (17), Prospective			Dupilumab	48wk	200mg/300mg^b^	Q2W/Q4W	6,y	13.4(3.1)[6-18],y	37.0(11.6)[NR]	NA	52	37
14	Amy S Paller et al, 2022 (18),RCT-Phase3			Dupilumab+TCS	16wk	200mg/300mg^b^	Q4W	6,m	4.2(NR)[0.5-6],y	35.1(13.9)[NR]	37.2(13.0)[NR]	NA	83
				Placebo+TCS			Q4W	6,m	3.8(NR)[0.5-6],y	33.1(12.2)[NR]	37.2(11.4)[NR]	NA	79
15	Lawrence Eichenfield et al, 2021 (19), RCT-Phase3			Abrocitinib	12wk	100mg	1time/d	12,y	16.0(NR)[14-17]	31.0 (12.8)[NR]	67.6 (13.5)[NR]	95	NA
				Abrocitinib	12wk	200mg	1time/d	12,y	15.0(NR)[13-16]	29.5 (12.2)[NR]	66.2 (13.3)[NR]	94	NA
				Placebo	12wk		1time/d	12,y	14.0(NR)[13.5-16.5]	29.2 (12.7)[NR]	68.5 (13.4)[NR]	96	NA
16	Shuba Rajashri Iyengar et al, 2018 (20), RCT-Phase2			Omalizumab	24wk	150-375mg	Q2W-4w	6,y	14.4 (NR)[NR]	NR	NR(NR)[37-100]	4(>4y)	NR
				Placebo		150-375mg	Q2W-4w	6,y	15.8(NR)[NR]	NR	NR(NR)[40-100]	4(>4y)	NR
17	Susan Chan et al, 2019 (21),RCT-Phase2			Omalizumab	24wk	150mg	Q4W	10,y	10.2 (0.1)N[NR]	45.5 (10.1)[NR]	55.5(9.5)[NR]	16(≥10y)	14 (<10y)
				Placebo		150mg	Q4W	10,y	10.4 (4.3)[NR]	43.5 (11.1)[NR]	54.3(7.7)[NR]	17(≥10y)	15 (<10y)
18	Mohamed A El-Khalawany et al, 2012 (22),Multicenter experience			Methotrexate	12wk	7.5 mg	QW	4,y	11.6 (1.52)[8-14]	NR	57.90 (3.21)[NR]	NR	20(<14y)
				Cyclosporine A		2.5 mg/kg	Qd	4,y	10.3 (2.82)[7-14]	NR	56.54 (4.82)[NR]	NR	20(<14y)
19	Bunikowski R et al, 2001 (23), Prospective			Cyclosporine A	8wk	2.5mg/kg	Bid	8,y	NR(NR)[1.83-15.75]	NR	61.7(NR)[NR]	NR	4(<16)
				Cyclosporine A	8wk	2.5mg/kg; 3.5mg/kg^c^	Bid	8,y	NR(NR)[1.83-15.75]	NR	61.7(NR)[NR]	NR	3(<16)
				Cyclosporine A	8wk	2.5mg/kg; 3.5mg/kg; 4.5mg;5mg/kg^c^	Bid	8,y	NR(NR)[1.83-15.75]	NR	61.7(NR)[NR]	NR	3(<16)
20	Jing-Long Huang et al, 2000 (24), Clinic trial			IVIG	24wk	2g/kg/dose	3times/month	7,m	9.4(2.3 )[7-12],m	NA	73(NR)[NR]	NA	5
				Control		2g/kg/dose	3times/month	7,m	9.2(2.1)[7-12],m	NA	74(NR)[NR]	NA	7
				Normal		2g/kg/dose	3times/month	7,m	9.5(2.1) [7-12],m	NA	NR	NA	10
21	Antonio Torrelo et al, 2023 (25), RCT-Phase3			Baricitinib+TCS	16wk	1mg	Qd	2,y	12.4 (4.1)[2-18]	26.6 (10.0)[NR]	63.6 (12.7)[NR]	121(<18y)	NR
						2mg	Qd	2,y	11.8 (3.7)[2-18]	26.8 (9.0)[NR]	62.4 (11.8)[NR]	120(<18y)	NR
						4mg	Qd	2,y	11.9 (3.8)[2-18]	25.3 (9.5)[NR]	60.7 (13.1)[NR]	120(<18y)	NR
				Placebo+TCS			Qd	2,y	11.8 (4.0)[2-18]	27.0 (10.3)[NR]	61.5 (11.9)[NR]	122(<18y)	NR
22	Amy S. Paller et al, 2023 (26),Upadacitinib, RCT-Phase3^d^,Measure Up 1			Upadacitinib	16wk	15mg	Qd	12,y	15.4 (2.0)[12-18]	30.9 (12.8)[NR]	NR	63	NR
	MeasureUp 2			Upadacitinib		15mg	Qd	12,y	15.2(1.8)[12-18]	28.0 (12.2)[NR]	NR	58	NR
	AD Up			Upadacitinib		15mg	Qd	12,y	15.4(1.7)[12-18]	29.6 (11.7)[NR]	NR	60	NR
	Measure Up 1			Upadacitinib	16wk	30mg	Qd	12,y	15.6(1.7)[12-18]	28.7 (10.7)[NR]	NR	58	NR
	MeasureUp 2			Upadacitinib		30mg	Qd	12,y	15.8(1.7)[12-18]	31.2 (14.0)[NR]	NR	62	NR
	AD Up			Upadacitinib		30mg	Qd	12,y	15.3(1.9)[12-18]	28.7 (10.1)[NR]	NR	60	NR
	Measure Up 1			Placebo			Qd	12,y	15.1(1.7)[12-18]	30.2 (14.2)[NR]	NR	58	NR
	MeasureUp 2			Placebo			Qd	12,y	15.5(1.7)[12-18]	30.1 (13.3)[NR]	NR	60	NR
	AD Up			Placebo			Qd	12,y	15.1(1.9)[12-18]	30.3 (12.1)[NR]	NR	63	NR
23	Amy S. Paller et al, 2023 (27),Tralokinumab, RCT-Phase3^d^			Tralokinumab	16wk	150mg	Q2W	12,y	NR(NR)[13-16]	NR(NR)[21.4-39.4]	NR(NR)[57.8-77.6]	98	NR
						300mg	Q2W	12,y	NR(NR)[13-16]	NR(NR)[21.1-37.8]	NR(NR)[59.4-75.6]	97	NR
				Placebo			Q2W	12,y	NR(NR)[13-16]	NR(NR)[19.7-35.8]	NR(NR)[57.8-76.7]	94	NR
24	Amy S. Paller et al, 2023 (27),Tralokinumab, RCT-Phase3, OLE^d^	98/NR/65	52wk	Tralokinumab	16wk	150mg	Q2W/Q4W	12,y	NR(NR)[13-16]	NR	NR	65	NR
		97/NR/70				300mg	Q2W/Q4W	12,y	NR(NR)[13-16]	NR	NR	70	NR
		94/NR/79		Placebo				12,y	NR(NR)[13-16]	NR	NR	79	NR

QW, quaque week; Q2W, quaque two weeks; Q4W, quaque quattuor weeks; Bid, bis in die; Qd, quaque die; EASI, Eczema Area and Severity Index; SCORAD, Scoring Atopic Dermatitis; NR, not reported; NA, not applicable; OLE,open-label extension study; RCT, randomized clinical trial; TCS, Topical Corticosteroid; IVIG, Intravenous Immunoglobulin;

a Results are listed per antiatopic agent; therefore, articles containing results on multiple treatment modalities are mentioned more than once.

b For Dupilumab, those under 60 kg receive a 400 mg loading dose (2 x 200 mg injections), followed by 200 mg every 2 weeks. Those weighing 60 kg or more receive a 600 mg loading dose (2 x 300 mg injections), followed by 300 mg every 2 weeks.

c The dosing regimen for Cyclosporine A is initiated with a starting dose of 2.5 mg/kg/day for all patients. Non-responders undergo a gradual dose escalation to a maximum of 5 mg/kg/day.

d The first authors and publication years of the two articles are identical (Amy S. Paller et al, 2023). To distinguish between them, distinct identifiers 'Upadacitinib' and 'Tralokinumab' are used, representing the full titles of the respective articles: 'Efficacy and Safety of Upadacitinib Treatment in Adolescents With Moderate-to-Severe Atopic Dermatitis: Analysis of the Measure Up 1, Measure Up 2, and AD Up Randomized Clinical Trials' and 'Efficacy and Safety of Tralokinumab in Adolescents With Moderate to Severe Atopic Dermatitis: The Phase 3 ECZTRA 6 Randomized Clinical Trial'.

**Table 2:** Study outcomes, quality, and risk of bias^a^.

	Study characteristic		Outcomes<52wk(4/12/16/24/36)		Outcomes>=52wk		Overall quality^d^/RoB^e^
	Source and Study design	Treatment	Patients aged >=12y	Patients aged <12y	Patients aged >=12y	Patients aged <12y	
1	Eric L. Simpson et al, 2019 (9),RCT-Phase3	Dupilumab 200mg Q2W^b^	Wk16:EASI-50 50 (61.0%) EASI-75 34(41.5%) EASI-90 19 (23.2%)SCORAD change:−51.6(3.2)	NA	NA	NA	A/7
		Dupilumab 300mg Q2W^b^		NA	NA	NA	A/7
		Dupilumab 300mg Q4W^b^	Wk16:EASI-50 46 (54.8%) EASI-75 32(38.1%) EASI-90 16 (19.0%)SCORAD change:−47.5(3.2)	NA	NA	NA	A/7
		Placebo	Wk16:EASI-50 11 (12.9) EASI-75 7 (8.2%) EASI-90 2 (2.4%) SCORAD change:−17.6(3.8)	NA	NA	NA	A/7
2	Andrew Blauvelt et al, 2022 (10),RCT-Phase3, OLE	Dupilumab 200/300mg Q2W;300mg Q4W^b^	NA	NA	Wk52:EASI-50 94 (93.1%)[NR]EASI-75 82(81.2%)EASI-90 57(56.4%)SCORAD change:−65.0(21.3)[NR]	NA	A/6
3	M.J. Cork et al, 2022 (11),RCT-Phase2	Dupilumab 2mg/kg QW	Wk12:EASI-50 14 (70%) EASI-75 11(55%) EASI-90 4(20%)SCORAD change:−48 (27)	NA	NA	NA	A/6
		Dupilumab 4mg/kg QW	Wk12:EASI-50 15 (75%) EASI-75 8(40%) EASI-90 5(25%)SCORAD change:−43(25)	NA	NA	NA	A/6
4	M.J. Cork et al, 2022 (11),RCT-Phase3, OLE	Dupilumab 2mg/kg QW	Wk16:EASI-50 17(100%) EASI-75 12(71%) EASI-90 6(35%)SCORAD change:−61(18)	NA	Wk52:EASI-50 16(100%) EASI-75 14(88%) EASI-90 7(44%)SCORAD change:−63 (19)	NA	A/4
		Dupilumab 4mg/kg QW	Wk16:EASI-50 18(95%) EASI-75 13(68%) EASI-90 10(53%)SCORAD change:−67(24)	NA	Wk52:EASI-50 16(89%) EASI-75 14(78%) EASI-90 11(61%)SCORAD change:−66 (26)	NA	A/4
5	A.S. Paller et al, 2020 (12),RCT-Phase2, OLE	Dupilumab 3mg/kg Single Injection	NA	Wk4:EASI-50 2 (20.0%) EASI-75 4(40.0%)SCORAD change: -22.4(42.52)	NA	NA	A/4
		Dupilumab 6mg/kg Single Injection	NA	Wk4:EASI-50 4 (40.0%) EASI-75 3(30.0%)SCORAD change: -43.2(35.55)	NA	NA	A/4
		Dupilumab 3mg/kg Single Injection	NA	Wk4:EASI-50 3 (30.0%) EASI-75 5(50.0%)SCORAD change: -26.6 (47.37)	NA	NA	A/4
		Dupilumab 6mg/kg Single Injection	NA	Wk4:EASI-50 4 (40.0%) EASI-75 5(50.0%)SCORAD change: -48.7(28.89)	NA	NA	A/4
6	M.J. Cork et al, 2020 (13),RCT-Phase2	Dupilumab 2mg/kg Single dose,QW	NA	Wk12:EASI-50 14 (78.0%) EASI-75 10(56.0%) EASI-90 6(33.0%)SCORAD change:−58(23)	NA	NA	A/6
		Dupilumab 4mg/kg Single dose,QW	NA	Wk12:EASI-50 11 (58.0%) EASI-75 9(47.0%) EASI-90 5(26.0%)SCORAD change:−47(24)	NA	NA	A/6
7	M.J. Cork et al, 2020 (13), RCT-Phase3, OLE	Dupilumab 2mg/kg QW	NA	Wk16:EASI-50 16 (94.0%) EASI-75 10(59.0%) EASI-90 7(41.0%)SCORAD change:−61 (31)	NA	Wk52:EASI-50 16 (94.0%) EASI-75 16 (94.0%) EASI-90 12(71.0%)SCORAD change:−79(16)	A/4
		Dupilumab 4mg/kg QW	NA	Wk16:EASI-50 14 (93.0%) EASI-75 11(73.0%) EASI-90 5(33.0%)SCORAD change:−62(18)	NA	Wk52:EASI-50 15 (94.0%) EASI-75 12 (75.0%) EASI-90 7(44.0%)SCORAD change:−67(19)	A/4
8	Andreas WOLLENBERG et al, 2020 (10),RCT-Phase3	Dupilumab 300 mg+TCS Q4W^b^	NA	Wk16:EASI-50 111 (91.0%) EASI-75 85 (69.7%) EASI-90 51 (41.8%) SCORAD change -62.4(2.1)	NA	NA	A/6
		Dupilumab 200 mg+TCS Q2W^b^	NA	Wk16:EASI-50 51 (86.4%) EASI-75 44 (74.6%) EASI-90 21 (35.6%) SCORAD change -62.7(3.1)	NA	NA	A/6
		Placebo+TCS	NA	Wk16:EASI-50 53 (43.1%) EASI-75 33 (26.8%) EASI-90 9 (7.3%) SCORAD change -29.8(2.3)	NA	NA	A/6
9	Andreas WOLLENBERG et al, 2020 (10),RCT-Phase3, OLE	OLE Dupilumab 300 mg(+TCS) Q4W^b^	NA	Wk16:SCORAD change:-18.3(NR)	NA	Wk52:SCORAD change:-12.8(NR)[NR]	A/4
10	Andrew Blauvelt et al, 2020 (14), OLE	Dupilumab 2mg/kg or4mg/kg QW	NA	NA	Wk52:EASI-75 84(80.8%)	NA	B/3
11	L. Stingeni et al, 2020 (15), Prospective	Dupilumab 200/300mg Q2W^b^	Wk16:EASI-50 137(99.3%) EASI-75 89(64.5%)EASI-90 46(33.3%)	NA	NA	NA	A/5
12	L. Stingeni et al, 2022 (16), Prospective	Dupilumab 200/300mg Q2W^b^	NA	NA	Wk52:EASI-50 119 (98.3%) EASI-75 110 (91.0%) EASI-90 89(73.6%)	NA	B/5
13	Angel D. Paganet al, 2022 (17), Prospective	Dupilumab 200mg/300mg Q2W^b^	Wk24:EASI-50 NR(74.0%) EASI-75 NR(46.6%) EASI-90 NR(12.3%)	NA	NR	NR	B/4
14	Amy S Paller et al, 2022 (18),RCT-Phase3	Dupilumab 200 mg /300mg^b^+TCS	NA	Wk16:EASI-50 57 (69%) EASI-75 44 (53%) EASI-90 21 (25%)SCORAD change:−54.7(3.4)	NA	NA	B/4
		Placebo+TCS	NA	Wk16:EASI-50 16(20%)EASI-75 8 (11%) EASI-90 2 (3%)SCORAD change:−16.2(3.5)	NA	NA	B/4
15	Lawrence Eichenfield et al, (19), 2021,RCT-Phase3	Abrocitinib 100mg	Wk12:EASI-75 67 (72.0%) SCORAD-75 30(36.7%)	NA	NA	NA	A /7
		Abrocitinib 200mg	Wk12:EASI-75 61 (68.5%) SCORAD-75 32(34.8%)	NA	NA	NA	A /7
		Placebo	Wk12:EASI-75 39 (41.5%) SCORAD-75 12(12.9%)	NA	NA	NA	A /7
16	Shuba Rajashri Iyengar et al, 2018 (20),RCT-Phase2	Omalizumab 150-375mg Q2W-4w	Wk24:SCORAD change:-NR(NR)(20-50%)	NA	NA	NA	B/4
		Placebo 150-375mg Q2W-4w	Wk24:SCORAD change:-NR(NR)(45-80%)	NA	NA	NA	B/4
17	Susan Chan et al, 2019 (21),RCT-Phase2	Omalizumab 150mg Q4W	Wk24:SCORAD change:-12.4(12.1)	NA	NA	NA	A/7
		Placebo	Wk24:SCORAD change:-5.1(11.7)	NA	NA	NA	A/7
18	Mohamed A El-Khalawany et al, 2012 (22),Multicenter experience	Methotrexate 7.5 mg QW	NA	Wk12:SCORAD change:-26.25(7.03) Wk36:-24.90(10.88)	NA	NA	A/6
		Cyclosporine A 2.5 mg/kg Qd	NA	Wk12:SCORAD change:-25.01(8.21) Wk36:-21.01(10.91)	NA	NA	A/6
19	Bunikowski R et al, 2001 (23), Prospective	Cyclosporine A2.5mg/kg Bid	NR	Wk12:SCORAD change:-23.0(NR)[NR]	NA	NA	B/6
		Cyclosporine A 2.5mg/kg;3.5mg/kg Bid^c^	NR	Wk12:SCORAD change:-23.1(NR)	NA	NA	B/6
		Cyclosporine A 2.5mg/kg;3.5mg/kg;4.5mg;5mg/kg Bid^c^	NR	Wk12:SCORAD change:-23.2(NR)	NA	NA	B/6
20	Jing-Long Huang et al, 2000 (24),Clinic trial	IVIG 2g/kg/dose 3times/month	NA	Wk4:SCORAD change:-34.4(NR)[NR]	NA	Wk24:SCORAD change:-47.12(NR)[NR]	B/5
		Control	NA	Wk4:SCORAD change:-3.00(NR)[NR]	NA	Wk24:SCORAD change:-4.05(NR)[NR]	B/5
21	Antonio Torrelo et al, 2023 (25),RCT-Phase3	Baricitinib+TCS 1mg/d	Wk16: EASI-75 39 (32.2%) EASI-90 14 (11.6%)	NA	NA	NA	A/7
		2mg	Wk16: EASI-75 48 (40.0%)EASI-90 26 (21.7%)	NA	NA	NA	A/7
		4mg	Wk16: EASI-75 63 (52.5%)EASI-90 36 (30.0%)	NA	NA	NA	A/7
		Placebo+TCS	Wk16: EASI-75 39 (32.0%) EASI-90 15 (12.3%)	NA	NA	NA	A/7
22	Amy S. Paller et al, 2023 (26),Upadacitinib,RCT-Phase3^f^	Upadacitinib 15mg Measure Up 1 qd	Wk16: EASI-75 47(75%) EASI-90 30 (48%)	NA	NA	NA	A/7
		Upadacitinib 30mg Measure Up 1	Wk16: EASI-75 49 (85%)EASI-90 43 (74%)	NA	NA	NA	A/7
		Placebo	Wk16: EASI-75 7(12%)EASI-90 2(3%)	NA	NA	NA	A/7
		Upadacitinib 15mg MeasureUp 2	Wk16: EASI-75 40(69%) EASI-90 28(48%)	NA	NA	NA	A/7
		Upadacitinib 30mg MeasureUp 2	Wk16: EASI-75 46(73%)EASI-90 38(62%)	NA	NA	NA	A/7
		Placebo	Wk16: EASI-75 8(13%)EASI-90 1(2%)	NA	NA	NA	A/7
		AD Up	Wk16: EASI-75 38(63%) EASI-90 29(48%)	NA	NA	NA	A/7
		AD Up	Wk16: EASI-75 51(84%)EASI-90 44(74%)	NA	NA	NA	A/7
		Placebo	Wk16: EASI-75 19(30%)EASI-90 13(21%)	NA	NA	NA	A/7
23	Amy S. Paller et al, 2023 (27),Tralokinumab,RCT-Phase3^f^	Tralokinumab 150mg Q2W	Wk16: EASI-75 28(28.6%) EASI-90 19(19.4%) SCORAD decline:27.5(NR)	NA	NA	NA	A/7
		Tralokinumab 300mg Q2W	Wk16: EASI-75 27(27.8%)EASI-90 17(17.5%) SCORAD decline:29.1(NR)	NA	NA	NA	A/7
		Placebo	Wk16: EASI-75 6(6.4%)EASI-90 4(4.3%) SCORAD decline:9.5(NR)	NA	NA	NA	A/7
24	Amy S. Paller et al, 2023 (27),Tralokinumab,RCT-Phase3,OLE^f^	Tralokinumab 150mg Q2W	NA	NA	Wk52:EASI-50 NR(75.4%) EASI-90 NR(43.0%)	NA	A/4
		Tralokinumab 300mg Q2W	NA	NA	Wk52:EASI-50 NR(71.4%) EASI-90 NR(32.9%)	NA	A/4
		Placebo	NA	NA	Wk52:EASI-50 NR(70.9%) EASI-90 NR(43.1%)	NA	A/4

QW, quaque week; Q2W, quaque two weeks; Q4W, quaque quattuor weeks; Bid, bis in die; Qd, quaque die; EASI, Eczema Area and Severity Index; EASI50, 50% reduction in EASI; EASI75, 75%reduction in EASI; EASI90, 90% reduction in EASI; SCORAD, Scoring Atopic Dermatitis; NR, not reported; NA, not applicable; OLE, open-label extension study; RCT, randomized clinical trial; TCS,Topical Corticosteroid; IVIG, Intravenous Immunoglobulin.

^a^Results are listed per antiatopic agent; therefore, articles containing results on multiple treatment modalities are mentioned more than once.

^b^For Dupilumab, those under 60 kg receive a 400 mg loading dose (2 x 200 mg injections), followed by 200 mg every 2 weeks. Those weighing 60 kg or more receive a 600 mg loading dose (2 x 300 mg injections), followed by 300 mg every 2 weeks.

^c^The dosing regimen for Cyclosporine A is initiated with a starting dose of 2.5 mg/kg/day for all patients. Non-responders undergo a gradual dose escalation to a maximum of 5 mg/kg/day.

^d^The quality assessment of studies followed the Strengthening the Reporting of Observational Studies criteria for observational studies (6)and adhered to the Consolidated Standards of Reporting Trials statement for randomized trials (7). An "A" rating indicated that more than 80% of the criteria were met; "B" denoted 50% to 80% criteria fulfillment, while "C" indicated less than 50% criteria fulfillment.

^e^Evaluation of bias risk involved the utilization of the Newcastle-Ottawa Scale (NOS) (8) for cohort studies and the Cochrane Risk of Bias Tool for randomized studies (4). Additional details can be found in **Supplementary eTables 3** and **4**.

^f^The first authors and publication years of the two articles are identical (Amy S. Paller et al, 2023). To distinguish between them, distinct identifiers 'Upadacitinib' and 'Tralokinumab' are used, representing the full titles of the respective articles:

'Efficacy and Safety of Upadacitinib Treatment in Adolescents With Moderate-to-Severe Atopic Dermatitis: Analysis of the Measure Up 1, Measure Up 2, and AD Up Randomized Clinical Trials' and 'Efficacy and Safety of Tralokinumab in Adolescents With Moderate to Severe Atopic Dermatitis: The Phase 3 ECZTRA 6 Randomized Clinical Trial'.

### Dupilumab

A total of 10 studies (9–18) assessed the efficacy, safety, and tolerability of Dupilumab in a cumulative cohort of 1,675 pediatric and adolescent atopic dermatitis patients.

### Dupilumab efficacy and safety in infants and young children

For children aged 6 months to 6 years, in terms of efficacy, at the fourth week after a single injection of Dupilumab at doses of 3mg/kg or 6mg/kg, 30%-40% of patients aged 6 months to 2 years achieved EASI-75, while 50% of patients aged 2 to 6 years achieved EASI-75. In the group receiving a single injection of Dupilumab at a dose of 3 mg/kg, there was a change in SCORAD scores of -26.6%, whereas the group receiving a single injection of Dupilumab at a dose of 6 mg/kg exhibited a more substantial change with SCORAD scores decreasing by -48.7% (13). By the 16th week of receiving Dupilumab in combination with topical corticosteroids (TCS), there was a change in SCORAD scores of −54.7%,and 53% of patients aged 6 months to 2 years achieved EASI-75 (9) in the **Figures 2** and **3**. There were no efficacy data available beyond this time point. Regarding the safety profile of the drug, the most common adverse events observed were skin infections and dermatitis atopic (PT, preferred term) (9, 13).

### Dupilumab efficacy and safety in children aged 6 to 12 years

For children aged 6 to 12 years, in terms of efficacy, during weeks 12 to 16, with a dosage of Dupilumab at 2mg/kg, 56.0% to 59.0% of patients achieved EASI-75, and 33.0% to 41.0% of patients achieved EASI-90. With a dosage of Dupilumab at 4mg/kg, 33.0% to 47.0% of patients achieved EASI-75, and 26.0% to 33.0% of patients achieved EASI-90.Meanwhile, the percentage change in SCORAD scores ranged from -58% to -62% (14). At week 16 while receiving both Dupilumab and topical corticosteroids (TCS), 74.6% of patients receiving 200mg Dupilumab achieved EASI-75 (11) (**Figure 3**), and 69.7% of patients receiving 300mg Dupilumab achieved EASI-75, The percentage change in SCORAD scores for both dosage groups showed little difference, both around -62% (11) (**Figure 2**). In the long-term data, at week 52, patients receiving Dupilumab at a dosage of 2mg/kg had 94.0% achieving EASI-75, with 71.0% achieving EASI-90. Patients receiving Dupilumab at a dosage of 4mg/kg had 75.0% achieving EASI-75, with 44.0% achieving EASI-90. At this point, the SCORAD scores have shown a significant decrease compared to the 12-16 week range, ranging from approximately -67% to -79% (14). Overall, the incidence of treatment-emergent adverse events (TEAEs) observed in this study was relatively low, with the majority being of mild or moderate severity. In terms of safety, the most commonly observed adverse events were rhinitis and exacerbation of atopic dermatitis, followed by conjunctivitis, skin infections, and upper respiratory tract infections. The rate of severe adverse events was low, including occurrences such as patent ductus arteriosus, injection-site edema, food allergy, herpes simplex infection, and ankle fracture. Noteworthy adverse events included severe viral conjunctivitis, severe allergic conjunctivitis, mild atopic keratoconjunctivitis, mild suicidal ideation, and moderate depression. Two adverse events led to treatment discontinuation, comprising one case of moderate bilateral conjunctivitis and one case of moderate exacerbation of atopic dermatitis. (**Supplementary eTable 2**).

### Dupilumab efficacy and safety in adolescents

For adolescents aged 12 to 18 years, in terms of efficacy, during weeks 12 to 16, with a dosage of Dupilumab at 2mg/kg administered once weekly, 55% to 71% of patients achieved EASI-75, and 20% to 35% of patients achieved EASI-90. With a dosage of Dupilumab at 4mg/kg administered once weekly, 40% to 68% of patients achieved EASI-75, and 25% to 53% of patients achieved EASI-90 (12). Looking at long-term efficacy, by week 52, 80.8% of patients achieved EASI-75.At this point, the SCORAD scores are both around -65%, indicating a greater change compared to the 12-16 week period (15). The most common adverse event observed was nasopharyngitis, and other adverse events included dermatitis atopic (PT), skin infections, conjunctivitis, and headache. Patients receiving Dupilumab at dosages of 200/300mg and administered once every two weeks achieved EASI-75 in 41.5% to 64.5% of cases during weeks 12-16 (10, 16) (**Figure 3**). Patients receiving Dupilumab at a dosage of 300mg once every four weeks had 38.1% achieving EASI-75 (10). Looking at long-term data, at week 52, patients receiving Dupilumab at dosages of 200/300mg every two weeks and 300mg every four weeks had 81.2% achieving EASI-75, with 56.4% achieving EASI-90 (11). In terms of safety, compared to the placebo group, the overall incidence of adverse events with Dupilumab was slightly higher, but occurrences leading to treatment discontinuation were infrequent. The most common adverse events were nasopharyngitis and atopic dermatitis (PT), followed by conjunctivitis, skin infections, and upper respiratory tract infections. Some less common adverse events included flushing (3.6%), injection-site reaction (2.9%), fatigue (1.4%), diarrhea (1.4%), headache (0.7%), and herpes simplex (0.7%) (16) (**Supplementary eTable 2**).

### Abrocitinib

One study (19) evaluated the efficacy, safety, and tolerability of Abrocitinib in a cumulative cohort of 285 adolescent atopic dermatitis patients. At week 12, a greater proportion of patients treated with abrocitinib at doses of 200 mg and 100 mg achieved EASI-75, at rates of 72.0% and 68.5%, respectively, compared with 41.5% in the placebo group (p<0.01 for both dosage groups). Regarding safety, the incidence of adverse events during treatment was 62.8% in the 200 mg group, 56.8% in the 100 mg group, and 52.1% in the placebo group. The rates of discontinuation due to adverse drug reactions were similar across all groups, at 2.1% for the 200 mg group, 1.1% for the 100 mg group, and 2.1% for the placebo group. Although the percentage of adverse events reported was higher in the treatment groups than in the placebo group, the percentage of treatment discontinuation was comparable. This may suggest that the majority of adverse events were of mild to moderate severity and were appropriately managed.

### Omalizumab

Two studies (20, 21) assessed the efficacy of Omalizumab in a cumulative cohort of 70 children and adolescents aged 6 to 18 with atopic dermatitis, while one study (21)evaluated the safety and tolerability of Omalizumab in a cumulative cohort of 62 children and adolescents within the same age range. In terms of efficacy, In the 24-week study, the treatment group receiving omalizumab demonstrated a significant improvement in objective SCORAD index compared to the placebo group, with a mean difference of -6.9 (95% CI:-12.2 to -1.5; P = 0.01) (21). On the other hand, patients receiving different doses and frequencies of Omalizumab treatment ranging from 150-375mg every 2 to 4 weeks also showed clinical improvement (SCORAD change of approximately 20% - 50%). However, the changes observed in the Omalizumab treatment group were comparable to the clinical effects observed in the placebo group (SCORAD change of approximately 45% - 80%), and this study had a smaller sample size and greater heterogeneity (20). In terms of safety, in the Omalizumab group (21), 15 participants (50%) reported respiratory events, while in the placebo group, 25 participants (78%) reported respiratory events (RR 0.64; 95% CI, 0.43-0.96). This suggests that Omalizumab treatment may reduce the risk of respiratory events in patients. Other adverse events included dermatitis atopic (PT), and there was no significant difference in the incidence of atopic dermatitis (PT) between the Omalizumab group and the placebo group (21) (**Supplementary eTable 2**). Overall, the omalizumab treatment group showed a lower risk of adverse event frequency compared to placebo, and no significant differences were observed in terms of severe adverse events and adverse events of particular concern.

### Conventional therapy

#### Cyclosporine A and methotrexate

One study (22) assessed the efficacy and safety of Methotrexate and Cyclosporine A in a cumulative cohort of 40 children and adolescents aged 4 to 14 with atopic dermatitis. Another study (23) evaluated the efficacy of Cyclosporine A in a cumulative cohort of 62 children and adolescents aged 6 to 16 with atopic dermatitis. In the Methotrexate treatment group, the SCORAD change at week 12 was -26.25% with a standard deviation of 7.03 (**Figure 2**). At week 36, the SCORAD change was -24.90% with a standard deviation of 10.88. In the Cyclosporine treatment group, the SCORAD change at week 12 was -25.01% with a standard deviation of 8.21(**Figure 2**), and at week 36, the SCORAD change was -21.01% with a standard deviation of 10.91%. This indicates that both Methotrexate and Cyclosporine treatment regimens led to significant SCORAD changes at week 12, demonstrating efficacy in alleviating symptoms of atopic dermatitis. At week 36, Methotrexate still showed a SCORAD change, although the change was slightly smaller compared to week 12. On the other hand, Cyclosporine showed a larger SCORAD change at week 36 compared to week 12. This may suggest that Cyclosporine could potentially exhibit better sustained efficacy in long-term treatment. In terms of safety, the study indicated that both Methotrexate and Cyclosporine could lead to fatigue, but the incidence was higher with Cyclosporine (45% compared to 30%). This suggests that Cyclosporine may be more likely to cause fatigue symptoms. Common adverse events for Methotrexate included anemia, abnormal liver functions, and nausea and vomiting, while common adverse events for Cyclosporine included leukopenia, headache and anemia. Therefore, these two drugs may lead to different types of adverse events (**Supplementary eTable 2**).

The another study evaluating Cyclosporine A dosage (23), increasing the dosage from 2.5mg/kg to 3.5mg/kg resulted in a SCORAD score change of 0.1 units within 12 weeks (from -23.0 to -23.1). This may suggest that increasing the dosage has a minimal impact on symptom improvement. Further increasing the Cyclosporine A dosage (from 2.5mg/kg to 3.5mg/kg, then to 4.5mg/kg and 5mg/kg) did not lead to a greater change in SCORAD scores. The SCORAD score only decreased by 0.1 units within 12 weeks, from -23.1 to -23.2. This may indicate that increasing the Cyclosporine A dosage did not significantly enhance symptom improvement (**Figure 2**).

#### IVIG

Intravenous immunoglobulins represent a therapy used off-label in patients with atopic dermatitis. One study (24) assessed the efficacy of IVIG (Intravenous Immunoglobulin) in a cohort of 12 infants aged 7-12 months with atopic dermatitis. In the IVIG treatment group (IVIG 2g/kg 3 times/month), there was a change of 34.4% in the SCORAD percentage score at week 4. At week 24, the SCORAD percentage score decreased by 47.12%. In contrast, the control group showed smaller changes in SCORAD scores during the same time period, with changes of 3.00 and 4.05 scores, respectively. These findings suggest that IVIG treatment appears to be more effective than topical corticosteroid therapy for improving symptoms of atopic dermatitis. These findings suggest that IVIG treatment appears to be potentially more effective than topical corticosteroid therapy in rapidly ameliorating symptoms of atopic dermatitis.

#### Baricitinib

A study (25) assessed the efficacy and safety of three doses of baricitinib in combination with low-to-moderate potency topical corticosteroids in 483 adolescents aged 12 to 18 with moderate-to-severe atopic dermatitis (AD). At week 16, patients receiving the high dose equivalent of baricitinib (4 mg) showed a significant increase in the proportion achieving ≥ 75% improvement in Eczema Area and Severity Index (EASI-75) compared to placebo (P = 0.002). Regarding safety, there was no significant difference in the occurrence of treatment-emergent adverse events (TEAEs) between patients treated with baricitinib and those receiving placebo. Abdominal pain, acne, and headache were the most commonly reported adverse events. Few patients discontinued the study drug due to adverse events (1.6% placebo vs. 0.6% baricitinib-treated, exact P-value not provided), or experienced serious adverse events (SAEs; 4.1% placebo vs. 1.1% baricitinib-treated, exact P-value not provided). Adverse events of special interest included two cases of ocular herpes simplex (one SAE and one leading to discontinuation), and one suicide attempt (SAE).

#### Upadacitinib

A study (26) comprising three clinical trials, namely Measure Up 1, Measure Up 2, and AD Up, assessed the efficacy and safety of 15 mg or 30 mg doses of upadacitinib in 542 adolescents aged 12 to 17 with moderate-to-severe atopic dermatitis. In Measure Up 1, the proportion of adolescents achieving EASI-75 at week 16 was 75% in the Upadacitinib 15 mg group, with an EASI-90 proportion of 48%, while in the Upadacitinib 30 mg group, the EASI-75 proportion was 85%, with an EASI-90 proportion of 74%. In comparison, the EASI-75 proportion in the placebo group was only 12%, with an EASI-90 proportion of 3%. In Measure Up 2, the EASI-75 proportion in the Upadacitinib 15 mg group was 69%, with an EASI-90 proportion of 48%, while in the Upadacitinib 30 mg group, the EASI-75 proportion was 73%, with an EASI-90 proportion of 62%. In contrast, the EASI-75 proportion in the placebo group was 13%, with an EASI-90 proportion of 2%. In the AD Up trial, the EASI-75 proportion in the Upadacitinib 15 mg group was 63%, with an EASI-90 proportion of 48%, while in the Upadacitinib 30 mg group, the EASI-75 proportion was 84%, with an EASI-90 proportion of 74%. In comparison, the EASI-75 proportion in the placebo group was 30%, with an EASI-90 proportion of 21%. These data indicate that in all trials, adolescents receiving Upadacitinib 15 mg and 30 mg showed significantly higher proportions of EASI-75 and EASI-90 at week 16 compared to the placebo group (nominal P <.001). In terms of improvement, the Upadacitinib 30 mg group was slightly more effective than the 15 mg group in EASI-75 and EASI-90. In terms of safety, the most common treatment-related adverse events in adolescents receiving upadacitinib included acne, headache, upper respiratory tract infection, creatine phosphokinase level elevations, and nasopharyngitis. There were few cases of treatment discontinuation due to serious infections such as moderate acne, subcutaneous abscess, cellulitis, severe varicella infection, and herpes zoster ophthalmicus. Overall, the rate of adverse events in adolescents receiving upadacitinib treatment through week 16 was similar to that in adults.

#### Tralokinumab

A study (27) assessed the efficacy and safety of Tralokinumab at doses of 150mg or 300mg in 289 adolescents aged 12 to 17 years with moderate-to-severe AD. At week 16, the proportion of patients achieving EASI-75 was 28.6% for those treated with 150mg Tralokinumab and 27.8% for those treated with 300mg Tralokinumab, compared to 6.4% in the placebo group, with P-values < 0.001 for both doses. At week 52, the proportions of patients achieving EASI-90 were 57.8% for the 150mg Tralokinumab group and 32.9% for the 300mg Tralokinumab group, with over 50% of patients meeting the primary endpoint at week 16 maintaining the efficacy of Tralokinumab. In terms of safety, Tralokinumab was well tolerated, with the majority of adverse events being mild or moderate. Serious adverse events were rare, with only one treatment-related adverse event leading to treatment discontinuation (not considered related to treatment); the most common adverse events included upper respiratory tract infection, exacerbation of atopic dermatitis, injection site reaction, asthma, and headache. The occurrence of conjunctivitis as a special concern adverse event was low and similar between the two Tralokinumab dose groups and the placebo group. The types and frequencies of adverse events observed during the maintenance and open-label periods were similar to those observed during the initial period.

## Discussion

Atopic dermatitis (AD) is a chronic inflammatory skin condition with a higher prevalence among adolescent patients (28, 29). It negatively impacts the quality of life for both patients and their families (30) and is associated with issues such as poor academic performance, social problems, anxiety, and depression (31). While topical treatments can effectively manage mild AD, moderate to severe cases often require systemic therapy (32, 33). Managing moderate to severe AD in children and adolescents presents challenges due to patient-related factors and a lack of scientific guidance, as many clinical trials have excluded these populations. This systematic review aims to provide a literature review of the effectiveness and safety of systemic anti-atopic dermatitis treatments in patients with moderate to severe AD.

Dupilumab is a fully human VelocImmune-derived monoclonal antibody that functions by blocking the shared receptor subunit for interleukin (IL)-4 and IL-13, thereby inhibiting the signaling of these two cytokines and reducing type 2 inflammation associated with atopic dermatitis (AD) and related atopic diseases such as asthma, allergic rhinitis, food allergy, chronic rhinosinusitis with nasal polyps, and eosinophilic esophagitis (34, 35). IL-4/IL-13 cytokines serve as crucial mediators of type 2 diseases, including AD and its associated atopic conditions (36). In phase 3 trials, dupilumab has demonstrated significant improvements in the signs and symptoms of AD, including pruritus, anxiety, and depression, as well as enhancing patient quality of life. It exhibits good safety profile in patients with moderate to severe AD (37, 38).

Dupilumab monotherapy demonstrated significant efficacy in infants, toddlers, children, and adolescents with AD. Significant improvements in SCORAD scores were observed after 4 weeks of treatment in infants and toddlers aged 6 months to 6 years, with better results seen at the 6mg/kg dose. When combined with topical corticosteroids, significant SCORAD score improvements were observed after 16 weeks in children aged 6 months to 12 years. Long-term data showed sustained efficacy across different age groups, particularly in adolescents aged 12 to 18 years. Dupilumab exhibited significant efficacy and safety comparable to that in adult patients, with EASI identified as a robust indicator reflecting disease improvement. A biweekly dosing regimen stratified by body weight normalized exposure in patients weighing less than 60kg and those weighing more than 60kg, and Dupilumab was more effective than placebo in both subgroups. The results for patients weighing less than 60kg were at least comparable to those for patients weighing 60kg or more on all key efficacy measures. These data support the use of body-weight stratified doses. The use of Dupilumab was associated with an increase in some injection site reactions and conjunctivitis, but at the same time reduced the risk of non-herpetic skin infections and performed similarly in adolescents and adults in terms of safety.

Once-daily oral abrocitinib, a Janus kinase (JAK) 1–selective inhibitor that modulates the function of key cytokines that are involved in AD pathogenesis and pruritus (39) (40).Abrocitinib has shown good efficacy and safety in the treatment of adolescent patients with atopic dermatitis. Both 100mg and 200mg doses of Abrocitinib were effective in treating atopic dermatitis, and although there was little difference between the two groups in EASI-75, the 100mg dose group was slightly more effective than the 200mg dose group in SCORAD-75. Common adverse events include nausea and herpes infection, but the low incidence of these adverse events indicates that the drug is acceptable in terms of efficacy and safety.

Patients treated with Omalizumab had a percentage decrease in their SCORAD score at week 24. A treatment regimen of 150mg of Omalizumab every 4 weeks resulted in an approximately -12.4% change in SCORAD. Another study of Omalizumab at different doses and frequencies was similar to the placebo group, with no apparent advantage. The second study had a smaller sample size and greater heterogeneity, which may have affected the stability and reliability of the results. Therefore, in order to more accurately evaluate the efficacy of Omalizumab, larger and more consistent studies may be needed to confirm this finding and determine whether there are advantages of specific doses and frequencies for improving efficacy.

Omalizumab is designed to bind human IgE, limiting mast cell degranulation and inflammatory mediator release (41).As the largest trial of omalizumab in atopic dermatitis to date and the first to demonstrate positive clinical results, it found significant improvement in omalizumab in the treatment of severe atopic dermatitis and severe allergies in children, after 24 weeks of treatment, The mean difference in the target SCORAD index was significant between the treatment group and the control group. The effect of omalizumab may be more pronounced in pediatric patients, especially those with higher IgE levels. Overall, omalizumab demonstrated a good safety and efficacy profile in the treatment of atopic dermatitis, particularly in reducing the use of powerful topical corticosteroids and improving quality of life (21). However, another study evaluating Omalizumab at different doses and frequencies did not demonstrate a significant advantage over the placebo group, and this finding may be less stable and reliable due to the smaller sample size and greater heterogeneity in the second study (20).While there is some controversy regarding the improvement in SCORAD scores, the research indicates that Omalizumab reduces IgE levels in the body (21), improves patients’ quality of life, and may suggest Omalizumab as a treatment option for children with severe atopic dermatitis that is difficult to manage.

Baricitinib is an oral reversible and selective Janus kinase (JAK) 1/JAK2 inhibitor that inhibits pro-inflammatory factors such as thymus interstitium lymphoblastopoietin (42), IL-4, IL-5, IL-13, IL-22 and IL-31 in the pathogenesis of AD (43) (44) (45).Compared with placebo, use of Baricitinib at a 4 mg equivalent dose resulted in clinically significant and statistically significant improvements in disease range and clinical markers of skin inflammation at 16 weeks. These clinically significant results were achieved quickly after initiation of treatment. In terms of safety, the safety of Baricitinib in pediatric patients with moderate to severe AD is consistent with that observed in adults.

Upadacitinib (ABT-494) is a selective JAK1 inhibitor that selectively targets JAK1-dependent disease drivers such as IL-6 and ifn-γ while reducing the effect on reticular cells and natural killer (NK) cells (46). Adolescents aged 12 to 17 years who received 15mg and 30mg of Upadacitinib achieved significant advantages over placebo at all efficacy endpoints at week 16. These results for skin clearance and reduction of pruritus are consistent with those observed in adults. The proportion of adolescents with anxiety and depression symptoms who received upadacitinib also showed a significant increase in the proportion without anxiety and depression symptoms. In terms of safety, the safety of upadacitinib in adolescents aged 12 to 17 years is similar to that observed in adults aged 18 to 75 years.

Interleukin (IL) -13 is considered to be a key cytokine driving AD progression (47) (48) (49). (50)Tralokinumab is a universal IgG4 monoclonal antibody that binds IL-13 with high affinity and intervenes in the development of AD disease by directly blocking the IL-13 signaling pathway (51). Tralokinumab outperformed placebo on all primary and key secondary endpoints and improved key psychosocial and symptomatic effects of AD through week 16, with clinical responses maintained in most patients on long-term maintenance and without the use of any potent TCS. The frequency and type of adverse events with tralokinumab were similar or lower in adolescent patients compared to adult studies. In particular, the incidence of conjunctivitis was low at 16 weeks and did not increase during 52 weeks of treatment. In addition, tralokinumab treatment did not increase the incidence of acne, and the incidence of herpes simplex infections and nausea was low between tralokinumab and placebo. Overall, the efficacy and safety of tralokinumab in adolescents is consistent with that of adults.

In the Methotrexate treatment group, a SCORAD change of -26.25% was observed at week 12, while the Cyclosporine A group showed a SCORAD change of -25.01%. Patients treated with IVIG (Intravenous Immunoglobulin) exhibited a 34.4% change in SCORAD percentage scores at week 4, which further decreased by 47.12% at week 24. In summary, all three treatments (Methotrexate, Cyclosporine A, and IVIG) appear effective in reducing SCORAD scores and improving symptoms of atopic dermatitis in their respective treatment groups.Regarding traditional treatments, the only systemic drug approved by the U.S. Food and Drug Administration for pediatric atopic dermatitis is a systemic corticosteroid. Additionally, existing guidelines discourage the systemic use of corticosteroids (32, 33). Cyclosporine A and IVIG are both off-label treatments. Systemic immunosuppressants, such as cyclosporine, have been restricted for off-label use due to the potential for long-term adverse reactions. Therefore, traditional drugs should be used with caution. This implies that even though certain immunosuppressants may be effective in some cases, doctors need to exercise caution when considering their use and carefully weigh the potential risks and benefits.

## Limitation

The reliability of the multiple studies included may be limited by the small sample size. The data is too scarce and heterogeneous for proper meta-analysis, which limits the generalizability of the results. Results varied from study to study due to differences in dose, inclusion of biologic neophyte patients or patients previously exposed to biologics, concomitant medication, sample size, study design, and methodological approach.

## Conclusion

Existing research has shown that response to several systemic treatments is not affected by age. The incidence of adverse events was low. More data on the efficacy, effectiveness, and safety of systemic therapy in pediatric and adolescent patients is critical to optimize personalized, effective, and safe management of atopic dermatitis for this growing patient population.

## Data availability statement

The original contributions presented in the study are included in the article/**Supplementary Materials**, further inquiries can be directed to the corresponding author/s.

## Author contributions

YZ: Supervision, Writing – original draft, Writing – review & editing. RD: Methodology, Writing – review & editing. JB: Methodology, Writing – review & editing.
