# Supplementary material for: Effectiveness and safety of systemic therapy for moderate-to-severe atopic dermatitis in children and adolescent patients: a systematic review
Source: Front Immunol. 2024 May 15;15:1367099. doi: 10.3389/fimmu.2024.1367099 (PMC11133567; doi:10.3389/fimmu.2024.1367099)
Supplement: Supplementary file 1 [file Table_1.docx]

Supplementary Online Content

etable 1.Search strategy

etable 2. Summary of Included Studies on Safety of Systemic Therapies in Children and Adolescent Patients

etable 3. Risk of Bias Assessment of Included Studies According to the Newcastle-Ottawa Scale

etable 4. Risk of Bias Assessment of Included Studies According to the Cochrane Risk of Bias Tool

eReferences.

This supplementary material has been provided by the authors to give readers additional information about their work.

**etable 1.Search strategy**

| Principle | MeSH headings/free words for all systemic drugs AND "MeSH headings/free words for Atopic dermatitis" AND "child/adolescent MeSH headings/free words" |
| --- | --- |
| Systemic Drugs | H1 Antihistamines: Diphenhydramine, Hydroxyzine Systemic Antibiotics: Mupirocin Glucocorticoids Cyclosporine (CsA) Azathioprine (AZP) Mycophenolic acid (MPA) Methotrexate (MTX) Immunomodulators: Intravenous Immunoglobulins (IVIG), interferon-gamma Biologics: Rituximab, Omalizumab, Dupilumab, Lebrikizumab, Nemolizumab, Tralokinumab, Spesolimab Emerging Oral Systemic Agents: Baricitinib, Upadacitinib, Abrocitinib, Gusacitinib Traditional Chinese Medicine: Glycyrrhetinic acid, Doxepin hydrochloride, Thalidomide, Rehmannia |
| Content | ((((((((((((((((((((((((((((("Glucocorticoids"[Mesh] OR Glucocorticoid OR Glucocorticoid Effect OR Effect, Glucocorticoid OR Glucorticoid Effects OR Effects, Glucorticoid) OR ("Diphenhydramine"[Mesh]6. OR Benzhydramine OR Diphenylhydramine OR Diphenylhydramin OR 2-Diphenylmethoxy-N,N-dimethylethylamine OR Benhydramin OR Benadryl OR Benylin OR Diphenhydramine Citrate OR Citrate, Diphenhydramine OR Diphenhydramine Citrate (1:1) OR Diphenhydramine Hydrochloride OR Hydrochloride, Diphenhydramine OR Dormin OR Allerdryl OR Dimedrol)) OR ("Hydroxyzine"[Mesh] OR 2-(2-(4-((4-Chlorophenyl)phenylmethyl)-1-piperazinyl)ethoxy)ethanol OR Hydroxyzine Dihydrochloride OR Vistaril OR Hydroxyzine Pamoate OR Pamoate, Hydroxyzine OR Orgatrax OR Durrax OR Atarax OR Hydroxyzine Hydrochloride)) OR ("Mupirocin"[Mesh] OR Pseudomonic Acid A OR Mupirocin Calcium OR Mupirocin, Sodium Salt OR Mupirocin, Lithium Salt OR BRL-4910A OR BRL 4910A OR BRL4910A OR Mupirocin, 14C-Labeled OR 14C-Labeled Mupirocin OR Mupirocin, 14C Labeled OR Mupirocin, Calcium Salt (2:1) OR Mupirocin, Calcium Salt (2:1), Dihydrate OR Pseudomonic Acid OR Bactroban)) OR ("cyclosporin A, 4-(2-butenyl)-4,4,N-trimethylthreonine(1)-" [Supplementary Concept] OR MeBm2t)-CsA OR N-methyl-4-((E)-2-butenyl)-4,4-dimethylthreonine cyclosporin A OR MeBm(2)t1-CsA OR 4-(2-butenyl)-4,4,N-trimethyl-Tyr(1)-cyclosporin A OR (MeBm(2)t)(1)-CsA OR 4-(2-butenyl)-4,4,N-trimethyl-threonine(1)-cyclosporin A)) OR ("Azathioprine"[Mesh] OR Azothioprine OR Imurel OR Imuran OR Immuran OR Azathioprine Sodium OR Sodium, Azathioprine OR Azathioprine Sodium Salt OR Azathioprine Sulfate)) OR ("Mycophenolic Acid"[Mesh] OR Mycophenolate Mofetil OR Mofetil, Mycophenolate OR Mycophenolic Acid Morpholinoethyl Ester OR Cellcept OR Mycophenolate Sodium OR Sodium Mycophenolate OR Mycophenolate, Sodium OR Myfortic OR Mycophenolate Mofetil Hydrochloride OR Mofetil Hydrochloride, Mycophenolate OR RS 61443 OR RS-61443 OR RS61443)) OR ("Methotrexate"[Mesh] OR Amethopterin OR Methotrexate, (D)-Isomer OR Methotrexate, (DL)-Isomer OR Mexate OR Methotrexate Sodium OR Sodium, Methotrexate OR Methotrexate, Sodium Salt OR Methotrexate, Disodium Salt OR Methotrexate Hydrate OR Hydrate, Methotrexate OR Methotrexate, Dicesium Salt OR Dicesium Salt Methotrexate)) OR ("Immunoglobulins, Intravenous"[Mesh] OR Antibodies, Intravenous OR Intravenous Antibodies OR Immune Globulin, Intravenous OR Intravenous Immune Globulin OR Intravenous Immunoglobulins OR Intravenous IG OR IV Immunoglobulins OR Immunoglobulins, IV OR IVIG OR IV Immunoglobulin OR Immunoglobulin, IV OR Intravenous Immunoglobulin OR Immunoglobulin, Intravenous OR Flebogamma DIF OR Gamunex OR Globulin-N OR Globulin N OR Intraglobin OR Intraglobin F OR Intravenous Immunoglobulins, Human OR Human Intravenous Immunoglobulins OR Immunoglobulins, Human Intravenous OR Immune Globulin Intravenous (Human) OR Immunoglobulins, Intravenous, Human OR Human Intravenous Immunoglobulin OR Immunoglobulin, Human Intravenous OR Intravenous Immunoglobulin, Human OR Gammagard OR Gamimune OR Gamimmune OR Modified Immune Globulin (Anti-Echovirus Antibody) OR Privigen OR Sandoglobulin OR Venoglobulin OR Venoglobulin-I OR Venoglobulin I OR Venimmune OR Iveegam OR Alphaglobin OR Endobulin OR Gamimune N OR Gamimmune N OR Gammonativ)) OR ("Interferon-gamma"[Mesh] OR gamma-Interferon OR Interferon, Immune OR Immune Interferon OR Type II Interferon OR Interferon, Type II OR Interferon Type II OR Interferon, gamma)) OR ("Rituximab"[Mesh] OR CD20 Antibody, Rituximab OR Rituximab CD20 Antibody OR Mabthera OR IDEC-C2B8 Antibody OR IDEC C2B8 Antibody OR IDEC-C2B8 OR IDEC C2B8 OR GP2013 OR Rituxan)) OR ("Omalizumab"[Mesh] OR Xolair)) OR ("dupilumab" [Supplementary Concept] OR SAR231893 OR SAR-231893 OR Dupixent OR REGN668 OR REGN-668)) OR ("lebrikizumab" [Supplementary Concept] OR RO-5490255 OR RG-3637 OR TNX-650 OR MILR1444A OR MILR-1444A OR PRO301444 OR PRO-301444)) OR ("nemolizumab" [Supplementary Concept] OR CIM331 OR CIM-331)) OR ("tralokinumab" [Supplementary Concept] OR CAT-354)) OR ("itepekimab" [Supplementary Concept] OR REGN-3500 OR REGN3500 OR SAR-440340 OR SAR440340)) OR ("spesolimab" [Supplementary Concept] OR BI 655130 OR BI655130)) OR ("baricitinib" [Supplementary Concept] OR 3-azetidineacetonitrile, 1-(ethylsulfonyl)-3-(4-(7H-pyrrolo(2,3-d)pyrimidin-4-yl)-1H-pyrazol-1-yl)- OR INCB-28050 OR Olumiant OR baricitinib phosphate OR baricitinib phosphate salt OR 3-azetidineacetonitrile, 1-(ethylsulfonyl)-3-(4-(7H-pyrrolo(2,3-d)pyrimidin-4-yl)-1H-pyrazol-1-yl)-, phosphate (1:1) OR INCB028050 OR INCB-028050 OR LY3009104 OR LY-3009104)) OR ("upadacitinib" [Supplementary Concept] OR (3S,4R)-3-ethyl-4-(3H-imidazo(1,2-a)pyrrolo(2,3-e)pyrazin-8-yl)-N-(2,2,2-trifluoroethyl)-1-pyrrolidinecarboxamide OR ABT-494 OR Rinvoq)) OR ("abrocitinib" [Supplementary Concept] OR N-(cis-3-(Methyl(7H-pyrrolo(2,3-d)pyrimidin-4-yl)amino)cyclobutyl)-1-propanesulfonamide OR N-(cis-3-(Methyl(7H-pyrrolo(2,3-d)pyrimidin-4-yl)amino)cyclobutyl)-propane-1-sulfonamide OR 1-Propanesulfonamide, N-(cis-3-(methyl-7H-pyrrolo(2,3-d)pyrimidin-4-ylamino)cyclobutyl)- OR PF-04965842)) OR ("Glycyrrhetinic Acid"[Mesh] OR Acid, Glycyrrhetinic OR Glycyram OR Glycyrrhetic Acid OR Acid, Glycyrrhetic OR Glyciram OR Jintan OR Rhetinic Acid OR Acid, Rhetinic OR Uralenic Acid OR Acid, Uralenic OR Enoxolone OR Arthrodont OR Po 12 OR 12, Po)) OR ("Doxepin"[Mesh] OR Deptran OR Desidox OR Doneurin OR Doxepia OR Doxepin beta OR Doxepin Hydrochloride OR Hydrochloride, Doxepin OR Doxepin Hydrochloride, Cis-Trans Isomer Mixture (approximately 1:5) OR Doxepin-RPh OR Doxepin RPh OR Espadox OR Mareen OR Novo-Doxepin OR Novo Doxepin OR Prudoxin OR Quitaxon OR Sinequan OR Sinquan OR Zonalon OR Xepin OR Aponal OR Apo-Doxepin OR Apo Doxepin OR ApoDoxepin)) OR ("Thalidomide"[Mesh] OR Sedoval OR Thalomid)) OR ("Rehmannia"[Mesh] OR Rehmannia glutinosa OR Foxglove, Chinese OR Chinese Foxglove)) OR (itepekimab)) OR (gusacitinib)) OR (CsA AND (clinicaltrial[Filter] OR randomizedcontrolledtrial[Filter]))) AND ("Dermatitis, Atopic"[Mesh] OR Atopic Dermatitides OR Atopic Dermatitis OR Dermatitides, Atopic OR Neurodermatitis, Atopic OR Atopic Neurodermatitides OR Atopic Neurodermatitis OR Neurodermatitides, Atopic OR Neurodermatitis, Disseminated OR Disseminated Neurodermatitides OR Disseminated Neurodermatitis OR Neurodermatitides, Disseminated OR Eczema, Atopic OR Atopic Eczema OR Eczema, Infantile OR Infantile Eczema)) AND (("Child"[Mesh] OR Children) OR ("Adolescent"[Mesh] Adolescents OR Adolescence OR Teens OR Teen OR Teenagers OR Teenager OR Youth OR Youths OR Adolescents, Female OR Adolescent, Female OR Female Adolescent OR Female Adolescents OR Adolescents, Male OR Adolescent, Male OR Male Adolescent OR Male Adolescents OR)) |
| Result | 7540 EMBASE ；clinical trial or RCT：942  1163 PubMed ；clinical trial or RCT：267  249 Central； clinical trial or RCT：248 |

**etable 2. Summary of Included Studies on Safety of Systemic Therapies in Children and Adolescent Patients**


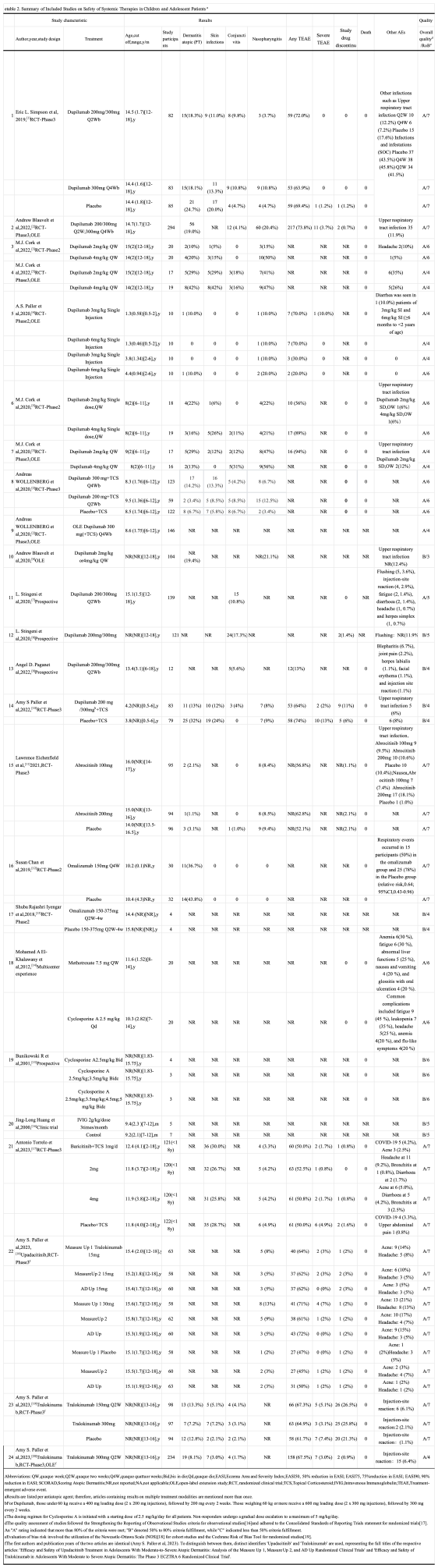


etable 3. Risk of Bias Assessment of Included Studies According to the Newcastle-Ottawa Scale

|  |  | Selection | | | | | Comparability Outcome of cohorts | Outcome | | |
| --- | --- | --- | --- | --- | --- | --- | --- | --- | --- | --- |
|  | Author, year | Adequacy case definition | Representativeness of exposed cohort | Seletion non exposed cohort | Ascertainment of exposure | Outcome not present at beginning of study |  | Assessment of Outcome | Follow-up duration | Follow-up Adequacy |
| 1 | Mohamed A El-Khalawany et al,2012,^[1]^Multicenter experience | * | _ | NA | * | * | NA | * | * | * |
| 2 | Bunikowski R et al,2001,^[2]^ Prospective | * | * | NA | * | * | NA | * | * | _ |
| 3 | Jing-Long Huang et al,2000,^[3]^Clinic trial | * | NA | NA | * | * | NA | * | * | _ |
| 4 | L. Stingeni et al,2020,^[4]^Prospective | * | * | NA | * | * | NA | * | _ | _ |
| 5 | L. Stingeni et al,2022,^[5]^ Prospective | * | * | NA | * | * | NA | * | _ | _ |
| 6 | Angel D. Paganet al,2022,^[6]^ Prospective | NA | * | NA | * | * | NA | * | _ | _ |
| 7 | Mohamed A El-Khalawany et al,2012,^[1]^ Multicenter experience | * | * | NA | * | * | NA | * | _ | _ |

etable 4. Risk of Bias Assessment of Included Studies According to the Cochrane Risk of Bias Tool

|  |  | Random sequence generation (selection bias) | Allocation concealment (selection bias) | Blinding of participants and personnel (performance bias) | Blinding of outcome assessment (detection bias) | Incomplete outcome data (attrition bias) | Selective reporting (reporting bias) | Other bias |
| --- | --- | --- | --- | --- | --- | --- | --- | --- |
|  | RCT |  |  |  |  |  |  |  |
| 1 | Eric L. Simpson et al, 2019,^[7]^  RCT-Phase3 | low risk | low risk | low risk | low risk | low risk | low risk | low risk |
| 2 | M.J. Cork et al,2022, ^[2]^RCT-Phase2 | low risk | low risk | unclear risk | low risk | low risk | low risk | low risk |
| 3 | Andreas WOLLENBERG et al,2020, ^[8]^RCT-Phase3 | low risk | low risk | low risk | low risk | low risk | low risk | low risk |
| 4 | Amy S Paller et al,2022,^[9]^ RCT-Phase3 | low risk | low risk | low risk | low risk | unclear risk | low risk | unclear risk |
| 5 | Lawrence Eichenfield et al,^[10]^ 2021,RCT-Phase3 | low risk | low risk | low risk | low risk | low risk | low risk | low risk |
| 6 | M.J. Cork et al,2020,^[11]^RCT-Phase2 | low risk | low risk | low risk | low risk | low risk | low risk | unclear risk |
| 7 | Susan Chan et al,2019,^[12]^RCT-Phase2 | low risk | low risk | low risk | low risk | low risk | low risk | low risk |
| 8 | Shuba Rajashri Iyengar et al,2018,^[13]^RCT-Phase2 | low risk | low risk | low risk | low risk | unclear risk | low risk | unclear risk |
| 9 | Antonio Torrelo et al,2023,^[14]^ RCT-Phase3 | low risk | low risk | low risk | low risk | low risk | low risk | low risk |
| 10 | Amy S. Paller et al,2023,^[15]^Upadacitinib,RCT-Phase3^a^ | low risk | low risk | low risk | low risk | low risk | low risk | low risk |
| 11 | Amy S. Paller et al,2023,^[16]^ Tralokinumab,RCT-Phase3^a^ | low risk | low risk | low risk | low risk | low risk | low risk | low risk |
|  | OLE |  |  |  |  |  |  |  |
| 1 | Andrew Blauvelt et al,2022,^[8]^ RCT-Phase3,OLE | low risk | high risk | high risk | high risk | low risk | low risk | low risk |
| 2 | M.J. Cork et al,2022,^[2]^ RCT-Phase3,OLE | low risk | high risk | high risk | high risk | low risk | low risk | low risk |
| 3 | A.S. Paller et al,2020,^[17]^RCT-Phase2,OLE | low risk | high risk | high risk | high risk | low risk | low risk | low risk |
| 4 | M.J. Cork et al,2020,^[11]^RCT-Phase3,OLE | low risk | high risk | high risk | high risk | low risk | low risk | low risk |
| 5 | Andreas WOLLENBERG et al,2020,^[8]^RCT-Phase3,OLE | low risk | high risk | high risk | high risk | low risk | low risk | low risk |
| 6 | Andrew Blauvelt et al,2020,^[12]^OLE | unclear risk | high risk | high risk | high risk | low risk | low risk | low risk |
| 7 | Amy S. Paller et al,2023,Tralokinumab,^[16]^RCT-Phase3,OLE^a^ | low risk | high risk | high risk | high risk | low risk | low risk | low risk |

OLE,open-label extension study;RCT, randomized clinical trial.

aThe first authors and publication years of the two articles are identical (Amy S. Paller et al, 2023). To distinguish between them, distinct identifiers 'Upadacitinib' and 'Tralokinumab' are used, representing the full titles of the respective articles: 'Efficacy and Safety of Upadacitinib Treatment in Adolescents With Moderate-to-Severe Atopic Dermatitis: Analysis of the Measure Up 1, Measure Up 2, and AD Up Randomized Clinical Trials' and 'Efficacy and Safety of Tralokinumab in Adolescents With Moderate to Severe Atopic Dermatitis: The Phase 3 ECZTRA 6 Randomized Clinical Trial'.

**eReferences.**

[1] El-Khalawany M A, Hassan H, Shaaban D, Ghonaim N, Eassa B. Methotrexate versus cyclosporine in the treatment of severe atopic dermatitis in children: a multicenter experience from Egypt.[J]. European journal of pediatrics, 2013, 172(3): 351–356.

[2] Cork M J, Thaçi D, Eichenfield L F, Arkwright P D, Hultsch T, Davis J D, Zhang Y, Zhu X, Chen Z, Li M, Ardeleanu M, Teper A, Akinlade B, Gadkari A, Eckert L, Kamal M A, Ruddy M, Graham N M H, Pirozzi G, Stahl N, DiCioccio A T, Bansal A. Dupilumab in adolescents with uncontrolled moderate-to-severe atopic dermatitis: results from a phase IIa open-label trial and subsequent phase III open-label extension.[J]. The British journal of dermatology, 2020, 182(1): 85–96.

[3] Huang J L, Lee W Y, Chen L C, Kuo M L, Hsieh K H. Changes of serum levels of interleukin-2, intercellular adhesion molecule-1, endothelial leukocyte adhesion molecule-1 and Th1 and Th2 cell in severe atopic dermatitis after intravenous immunoglobulin therapy.[J]. Annals of allergy, asthma & immunology : official publication of the American College of Allergy, Asthma, & Immunology, 2000, 84(3): 345–352.

[4] Stingeni L, Bianchi L, Antonelli E, Caroppo E S, Ferrucci S M, Ortoncelli M, Fabbrocini G, Nettis E, Schena D, Napolitano M, Gola M, Bonzano L, Rossi M, Belloni Fortina A, Balato A, Peris K, Foti C, Guarneri F, Romanelli M, Patruno C, Savoia P, Fargnoli M C, Russo F, Errichetti E, Bianchelli T, Bianchi L, Pellacani G, Feliciani C, Offidani A, Corazza M, Micali G, Milanesi N, Malara G, Chiricozzi A, Tramontana M, Hansel K. Moderate-to-severe atopic dermatitis in adolescents treated with dupilumab: A multicentre Italian real-world experience.[J]. Journal of the European Academy of Dermatology and Venereology : JEADV, 2022, 36(8): 1292–1299.

[5] Stingeni L, Bianchi L, Antonelli E, Caroppo E S, Ferrucci S M, Gurioli C, Ortoncelli M, Fabbrocini G, Nettis E, Schena D, Napolitano M, Gola M, Bonzano L, Rossi M, Belloni Fortina A, Balato A, Peris K, Foti C, Guarneri F, Romanelli M, Patruno C, Savoia P, Esposito M, Russo F, Errichetti E, Bianchelli T, Bianchi L, Pellacani G, Feliciani C, Offidani A, Corazza M, Micali G, Milanesi N, Malara G, Chiricozzi A, Tramontana M, Hansel K. A 52-week update of a multicentre Italian real-world experience on effectiveness and safety of dupilumab in adolescents with moderate-to-severe atopic dermatitis.[J]. Journal of the European Academy of Dermatology and Venereology : JEADV, 2023, 37(3): e384–e388.

[6] Pagan A D, David E, Ungar B, Ghalili S, He H, Guttman-Yassky E. Dupilumab Improves Clinical Scores in Children and Adolescents With Moderate to Severe Atopic Dermatitis: A Real-World, Single-Center Study.[J]. The journal of allergy and clinical immunology. In practice, 2022, 10(9): 2378–2385.

[7] Simpson E L, Paller A S, Siegfried E C, Boguniewicz M, Sher L, Gooderham M J, Beck L A, Guttman-Yassky E, Pariser D, Blauvelt A, Weisman J, Lockshin B, Hultsch T, Zhang Q, Kamal M A, Davis J D, Akinlade B, Staudinger H, Hamilton J D, Graham N M H, Pirozzi G, Gadkari A, Eckert L, Stahl N, Yancopoulos G D, Ruddy M, Bansal A. Efficacy and Safety of Dupilumab in Adolescents With Uncontrolled Moderate to Severe Atopic Dermatitis: A Phase 3 Randomized Clinical Trial.[J]. JAMA dermatology, 2020, 156(1): 44–56.

[8] Blauvelt A, Guttman-Yassky E, Paller A S, Simpson E L, Cork M J, Weisman J, Browning J, Soong W, Sun X, Chen Z, Kosloski M P, Kamal M A, Delevry D, Chuang C-C, O’Malley J T, Bansal A. Long-Term Efficacy and Safety of Dupilumab in Adolescents with Moderate-to-Severe Atopic Dermatitis: Results Through Week 52 from a Phase III Open-Label Extension Trial (LIBERTY AD PED-OLE).[J]. 2022, 23(3). ,2022.

[9] Paller A S, Simpson E L, Siegfried E C, Cork M J, Wollenberg A, Arkwright P D, Soong W, Gonzalez M E, Schneider L C, Sidbury R, Lockshin B, Meltzer S, Wang Z, Mannent L P, Amin N, Sun Y, Laws E, Akinlade B, Dillon M, Kosloski M P, Kamal M A, Dubost-Brama A, Patel N, Weinreich D M, Yancopoulos G D, O’Malley J T, Bansal A. Dupilumab in children aged 6 months to younger than 6 years with uncontrolled atopic dermatitis: a randomised, double-blind, placebo-controlled, phase 3 trial.[J]. Lancet (London, England), 2022, 400(10356): 908–919.

[10] Eichenfield L F, Flohr C, Sidbury R, Siegfried E, Szalai Z, Galus R, Yao Z, Takahashi H, Barbarot S, Feeney C, Zhang F, DiBonaventura M, Rojo R, Valdez H, Chan G. Efficacy and Safety of Abrocitinib in Combination With Topical Therapy in Adolescents With Moderate-to-Severe Atopic Dermatitis: The JADE TEEN Randomized Clinical Trial.[J]. JAMA dermatology, 2021, 157(10): 1165–1173.

[11] Cork M J, Thaçi D, Eichenfield L F, Arkwright P D, Sun X, Chen Z, Akinlade B, Boklage S, Guillemin I, Kosloski M P, Kamal M A, O’Malley J T, Patel N, Graham N M H, Bansal A. Dupilumab provides favourable long-term safety and efficacy in children aged ≥ 6 to < 12 years with uncontrolled severe atopic dermatitis: results from an open-label phase IIa study and subsequent phase III open-label extension study.[J]. The British journal of dermatology, 2021, 184(5): 857–870.

[12] Chan S, Cornelius V, Cro S, Harper J I, Lack G. Treatment Effect of Omalizumab on Severe Pediatric Atopic Dermatitis: The ADAPT Randomized Clinical Trial.[J]. JAMA pediatrics, 2020, 174(1): 29–37.

[13] Andreae D A, Wang J. Immunologic effects of omalizumab in children with severe refractory atopic dermatitis: a randomized, placebo-controlled clinical trial.[J]. Pediatrics, 2014, 134 Suppl 3: S160.

[14] Torrelo A, Rewerska B, Galimberti M, Paller A, Yang C-Y, Prakash A, Zhu D, Pontes Filho M A G, Wu W-S, Eichenfield L F. Efficacy and safety of baricitinib in combination with topical corticosteroids in paediatric patients with moderate-to-severe atopic dermatitis with an inadequate response to topical corticosteroids: results from a phase III, randomized, double-blind, placebo-controlled study (BREEZE-AD PEDS).[J]. The British journal of dermatology, 2023, 189(1): 23–32.

[15] Paller A S, Ladizinski B, Mendes-Bastos P, Siegfried E, Soong W, Prajapati V H, Lio P, Thyssen J P, Simpson E L, Platt A M, Raymundo E M, Liu J, Calimlim B M, Huang X, Gu Y, Hu X, Yang Y, Su J C, Zheng M, Yamamoto-Hanada K, Teixeira H D, Irvine A D. Efficacy and Safety of Upadacitinib Treatment in Adolescents With Moderate-to-Severe Atopic Dermatitis: Analysis of the Measure Up 1, Measure Up 2, and AD Up Randomized Clinical Trials.[J]. JAMA dermatology, 2023, 159(5): 526–535.

[16] Paller A S, Flohr C, Cork M, Bewley A, Blauvelt A, Hong H C-H, Imafuku S, Schuttelaar M L A, Simpson E L, Soong W, Arlert P, Lophaven K W, Kurbasic A, Soldbro L, Vest N S, Wollenberg A. Efficacy and Safety of Tralokinumab in Adolescents With Moderate to Severe Atopic Dermatitis: The Phase 3 ECZTRA 6 Randomized Clinical Trial.[J]. JAMA dermatology, 2023, 159(6): 596–605.

[17] Paller A S, Siegfried E C, Simpson E L, Cork M J, Lockshin B, Kosloski M P, Kamal M A, Davis J D, Sun X, Pirozzi G, Graham N M H, Gadkari A, Eckert L, Ruddy M, Bansal A. A phase 2, open-label study of single-dose dupilumab in children aged 6 months to <6 years with severe uncontrolled atopic dermatitis: pharmacokinetics, safety and efficacy.[J]. Journal of the European Academy of Dermatology and Venereology : JEADV, 2021, 35(2): 464–475.
